# ‘Bridging the Gap': Do Psychiatry Core Trainees Feel Prepared to Deal With Acute or Emergency Physical Health Conditions?

**DOI:** 10.1192/bjo.2022.130

**Published:** 2022-06-20

**Authors:** Sian Holdridge

**Affiliations:** South London and the Maudsley NHS Foundation Trust, London, United Kingdom

## Abstract

**Aims:**

In many cases there are several years between a trainee finishing foundation training and covering inpatient psychiatric wards on call. However, being resident on-call and covering psychiatric wards involves dealing with acute medical as well as psychiatric emergencies. Anecdotally trainees say that they often feel unprepared for this, and that the types of medical emergencies seen in psychiatric wards have rarely been come across in foundation training. The views of the psychiatric core trainees in the Maudsley Training Programme were audited with the aim of finding out how confident they feel in this area.

**Methods:**

30 Maudsley core trainees were sent a questionnaire which included questions such as “how confident do you feel when dealing with physical health issues, particularly when on call?” and “how rusty do you feel on your physical health medicine from med school?” using a Likert scale.

Following the results of this initial audit an intervention was introduced which was the delivery of a monthly 10 minute slot at local teaching called “Bite-Sized Medicine”. This was a 10 minute power-point presentation on acute physical health issues.

Post-intervention there was a re-audit. Trainees were sent another questionnaire (Likert scale) asking follow-up questions to determine if the intervention had improved their confidence.

This project was approved by the South London and the Maudsley Information Governance team and did not require ethical approval.

**Results:**

10 core trainees responded during the pre-intervention audit and 13 during the post-intervention audit.

Mann Whitney U tests were used to compare the means Q1vs Q3 (confidence), and Q2 vs. Q4 (rustiness) pre and post intervention.

Both were significant on this output (P < 0.0005).

This shows that there is a significant difference in the mean scores pre and post intervention, with the post-intervention scores being higher. This indicates that the intervention helped trainees to feel more confident and less rusty in terms of dealing with acute physical health issues.

**Conclusion:**

In this small survey, core psychiatry trainees expressed that the introduction of “Bite-Sized Medicine” was useful in helping them feel more confident and prepared when dealing with acute and emergency physical health issues. This is reflected in the statistical analysis, albeit with small sample sizes. Comments were made such as “there are a range of physical health issues that are common in psychiatric care that trainees won't have seen much of in their foundation training. This is a very useful intervention for bridging that gap”.

